# 

**Published:** 1888-01-21

**Authors:** 


CONTENTS.

**The Battle of the "Pathies"** - 277

**Words of Consolation.** Chapter LXIII.—The Sufferings of Christ - 278

**The National Pension Fund for Nurses and Hospital Officials** - 279

**Christmas at the Hospitals.**—By our Commissioner and Correspondents.—St. Thomas's.—British Home for Incurables.—South Devon Hospital - 280

**The Limits and Influences of a Nurse's Vocation.** By R. Brudenell Carter, F.R.C.S. - 281

**Virtuous Journalism** - 282

**Notes for Practitioners.** By Geo. W. Potter, M.D. - 283

**Notes and News** - 284

**Everybody's Page** - 286

**Annotations.**—An Inquiry Answered.—May a Layman do Good?—The Charity Dispensing Society - 287

**A Working-Class View of Our Hospitals.** By the Chairman of Workmen's Governors, Sunderland Infirmary - 288

**Rachel Challice's Vow.** By Lina Nevill, Author of "A Future on Trust," etc. - 289

**Scotch Physicians and Surgeons.** By C. G. Furley.—Sir James Y. Simpson - 291

**The Nursing Mirror.**—Examinations for the Uncertificated - 292

**The Editor's Letter Box** - 292

**Amusements with Profit for all Ages.**—For Men and Women.—Children's Corner, etc. - iv.

The average issue of "The Hospital" now exceeds **10,000 COPIES WEEKLY,** and the circulation is steadily increasing.

